# Relationship of carotid intima-media thickness and duration of vegetarian diet in Chinese male vegetarians

**DOI:** 10.1186/1743-7075-8-63

**Published:** 2011-09-19

**Authors:** Shu-Yu Yang, Hui-Jie Zhang, Su-Yun Sun, Li-Ying Wang, Bing Yan, Chang-Qin Liu, Wei Zhang, Xue-Jun Li

**Affiliations:** 1 Fujian Academy of Integrative Medicine, Fujian University of Traditional Chinese Medicine, 1 Hua-Tuo Road, Shang-Jie Town, Fuzhou, 350108, PR China; 2 Xiamen Diabetes Institute, Department of Diabetes, The First Affiliated Hospital of Xiamen University, 55 Zhen-Hai Road, Xiamen 361003, PR China

**Keywords:** vegetarian diet, IMT, duration of vegetarian diet

## Abstract

**Objective:**

Many studies have shown that vegetarian diet has beneficial effects on the prevention of cardiovascular diseases. However, the effect of vegetarian diet on carotid intima-media thickness (IMT), as well as the association between IMT and duration of vegetarian diet, are still unclear. The present study aims to investigate the influence of duration of vegetarian diet on cardiovascular risk factors, and more importantly on IMT among Chinese vegetarians.

**Methods:**

One hundred and seventy-one Chinese male vegetarians were screened for metabolic profile, cardiovascular risk and carotid IMT. They were compared with 129 age-matched omnivores recruited from a community-based health project. The effects of confounding factors were adjusted by stepwise logistic regression analysis.

**Results:**

Compared to the omnivores, the vegetarians had lower BMI, weight, systolic blood pressure and diastolic blood pressure. Also, the levels of triglyceride, total cholesterol, HDL-Cholesterol, LDL-Cholesterol, ApoA1, ApoB, uric acid, albumin and γ-glutamyltransferase were significantly reduced in vegetarians. Omnivores had significantly higher fasting blood glucose than that of vegetarians. However, there were no differences in fasting insulin, C-reactive protein and HOMA-IR between the two groups. IMT was thinner in the vegetarian group than in the omnivore group (0.59 ± 0.16 vs. 0.63 ± 0.10 cm, *P *< 0.05). The vegetarians were divided according to duration of vegetarian diet (< 6 years, 6 to ≤ 11 years, > 11 years), those in tertile 1 (< 6 years) and tertile 2 (6 to ≤ 11 years) had shown thinner IMT as compared to the omnivores, and tertile 3 had shown no reduction.

**Conclusion:**

A decrease in multiple cardiovascular risk factors such as BMI, blood pressure and lipid profile was associated with vegetarian diet. Moreover, taking a low-calorie, low-protein, or vegetarian diet might have great beneficial effects on IMT through improved lipid profile, and the beneficial effects appeared to be correlated with the duration of vegetarian diet.

## Introduction

The effects of vegetarian diets on the prevention of cardiovascular diseases (CVD) had been reported extensively. As a specific population which has successfully made long-term lifestyle modifications, the vegetarian diet could provide important insights into the potential efficacy of a diet in reducing cardiometabolic risk. Many studies have shown that vegetarians had both lower blood pressure and cardiovascular mortality as compared with omnivores [1-3], and the benefit was attributed to a more favorable lipid profile due to long-term low calorie low-protein vegetarian diet [4]. However, some others did not show the beneficial effects of vegetarian diet [5,6].

Carotid IMT has been shown to predict CVD and is able to provide a surrogate end point to assess early atherosclerosis [7,8]; however, whether vegetarian diet could improve IMT is unclear. Furthermore, the association of the duration of vegetarian diet and IMT is still not established. The purpose of the present study was to investigate the protective effects of vegetarian diet on cardiometabolic risk, such as IMT, lipid profile, blood pressure and insulin sensitivity, and the relationship between its beneficial effects and duration of vegetarian diet in Chinese male vegetarians.

## Subjects and methods

### Subjects

One hundred and seventy-one vegetarians (age range: 21-76 years, mean age: 32.6 ± 12.7) were recruited from Nanputuo Temple of Xiamen in China. They did not take meat or fish as according to their religious beliefs, but a small part would occasionally consumed eggs and milk. The participants were on vegetarian diet at least one year (mean 10.4 ± 8.0 years, range, 1-44 years). The control group included 129 matched healthy omnivorous men from a community-based health project in Xiamen; no vitamins were taken during the previous 6 months, and were not on a lipid-lowering or weight-loss diets. The major study exclusion criteria were as follows: body weight ≧ 120% of ideal, a history of chronic disease (renal disease, cancer, diabetes mellitus, heart disease, and hypertension), alcohol intake, and current cigarette smoking. All subjects signed informed consents, and the study protocol was approved by the Human Research Ethics Committee of the First Hospital Affiliated to Xiamen University.

### Study protocol

#### Dietary assessment

Subjects were interviewed and the average of three 24 h dietary recalls (two on week days and one on weekends) were used to estimate daily consumption of different nutrients. A database for Chinese food composition was used to calculate the daily energy and nutrient intake [9,10].

#### Clinical assessment and laboratory measurements

All subjects were screened for health status by means of questionnaires regarding past medical history, family history, dietary preferences, starting age and duration of vegetarian diet and personal data, such as smoking status, alcohol consumption, education level, and physical exercise status. After a 10 to 12 h overnight fasting, subjects received anthropometric and biochemical measurements. Anthropometric measurements include weight, height, waist circumference, blood pressure (BP) and body mass index (BMI).

Serum lipids, including triglyceride (TG), total cholesterol (TC), high-density lipoprotein cholesterol (HDL-C), apolipoprotein A1 (ApoA1) and apolipoprotein B (ApoB); including total protein (TP), albumin (ALB), γ-glutamyl transferase (γ-GT); homocysteine (HCY), uric acid (UA) were assayed by enzymatic methods with automatic multi-channel chemical analyzer (Hitachi 7450, Hitachi Corp., Tokyo, Japan). Low-density lipoprotein cholesterol (LDL-C) was calculated by Friedewald's formula. Fasting glucose was determined by the glucose oxidase method using a Monarch Chemistry 2000 Autoanalyzer (Instrumentation Laboratory, Oakbrook Terrace, IL, USA) through standard laboratory techniques. Serum fasting insulin concentrations were measured by electrochemiluminiscence immunoassay (Roche Elecsys Insulin Test, Roche Diagnostics, Mannheim, Germany). HOMA-insulin resistance (HOMA-IR) was calculated by fasting serum insulin (FIns, mU/ml) ×fasting blood glucose (FBG, mmol/l)/22.5. C-reactive protein (CRP) was assayed in one batch by use of an immunoassay method (BN ProSpec System, Dade Behring, Marburg, Germany).

#### Measurement of carotid artery IMT

All subjects underwent B-mode ultrasonography of the extracranial carotid arteries by the use of a duplex system (a high-resolution ultrasound instrument ATL, HDI 5000, Phillips Sistemi Medical, Bothell, WA, USA) with a 7- to 10- MHz linear array multi-frequency transducer). All the examinations were performed by the same ultrasonographer blinded to clinical information. The right and left common carotid arteries and internal carotid arteries (including bifurcations) were evaluated with the subjects in supine position, with the head turned away from the sonographer and the neck extended with mild rotation. The intima-media thickness (IMT), defined as the distance between the intimal-luminal interface and the medial-adventitial interface, was measured. Briefly, in posterior approach and with the sound beam set perpendicular to the arterial surface, 1 cm from the bifurcation, three longitudinal measurements of IMT were completed and the mean value of six measurements from right (IMT-R) and left (IMT-L) carotid arteries were used. The mean IMT was the average of the IMTs from the right and left carotid arteries. Plaque, detected in longitudinal and transverse planes with anterior, lateral, and posterior approaches, was defined as an echogenic focal structure encroaching the vessel lumen with a distinct area 50% greater than the IMT of neighboring sites. The average absolute difference and standard deviation between measurements for 30 participants who had replicate maximum IMT measurements was 0.02 ± 0.04 mm.

### Statistical analysis

Statistical analyses were performed with SAS, version 8.01. Data are presented as means ± standard deviation (S.D) or means (95% confidence interval). Data that were not normally distributed were logarithmically transformed before analysis. Unpaired Student's t-test (or Manne Whitney U-test) and chi-square test were used for testing differences for numerical and nominal variables. Theχ^2^-test was used to compare categorical variables between groups. The correlation of duration of vegetarian diet with metabolic parameters was analyzed by Pearson correlation and multivariate linear regression analysis. The vegetarians were classified into three tertiles according to the duration of vegetarian diet or age of starting vegetarian diet respectively. The differenced groups were compared using an ANOVA test. The definition of the upper normal limit of IMT was set at the 75 th upper percentile of the IMT distribution in controls [11]. Multiple logistic regression analysis was used to assess the odds ratio (OR) for the presence of thick IMT according to tertiles of duration of vegetarian diet and age of starting vegetarian diet. Two-sided values of *P*< 0.05 were considered significant.

## Results

### Clinical characteristics of subjects

The vegetarians consumed less energy, protein, and fat than the omnivores (Additional File 1 Table S1); this is consistent with the lower plasma concentrations of total protein, triglycerides and cholesterol in vegetarians. Total fiber and polyunsaturated fatty acids (PUFA) intakes were similar between the two groups, whereas relative saturated fatty acid (SFA) and monounsaturated fatty acids (MUFA) intake were significantly lower in the vegetarians.

Characteristics of all subjects are shown in Table 1 and there were no significant differences in age and waist circumference between the two groups. Compared to the omnivores, the vegetarians had lower BMI, weight, systolic BP and diastolic BP. Also, the levels of TG, TC, HDL-C, LDL-C, ApoA1, ApoB, UA, ALB and γ-GT were significantly reduced in vegetarians. Omnivores had significantly higher FBG than the vegetarians. However, there were no differences in fasting serum insulin, C-reactive protein and HOMA-IR between the two groups.

**Table 1:** Baseline characteristics of the subjects.

Variables	Vegetarians (n = 171)	Omnivores (n = 129)	*P*
Age (yrs)	32.6 ± 12.7	34.2 ± 6.0	0.134
Duration of vegetarian diet (yrs)	10.4 ± 8.0	-	-
Systolic BP (mmHg)	116.0 ± 14.0	126.0 ± 15.0	< 0.001^‡^
Diastolic BP (mmHg)	71.0 ± 11.0	79.0 ± 9.0	< 0.001^‡^
BMI (kg/m^2^)	23.6 ± 4.0	24.4 ± 2.7	0.037^†^
Weight (kg)	66.34 ± 13.27	71.42 ± 8.43	< 0.001^†^
Waist Circumference (cm)	81.7 ± 10.6	80.7 ± 6.9	0.299^†^
Waist to hip ratio	0.88 ± 0.06	0.86 ± 0.05	0.028^†^
Triglyceride (mg/dl)	1.13 ± 0.68	1.38 ± 0.87	0.004^‡^
Total cholesterol (mg/dl)	4.26 ± 0.63	5.01 ± 0.86	< 0.001^‡^
HDL-cholesterol (mg/dl)	1.12 ± 0.18	1.23 ± 0.22	< 0.001^‡^
LDL- cholesterol (mg/dl)	2.59 ± 0.56	3.11 ± 0.80	< 0.001^‡^
ApoA_1 _(g/L)	1.06 ± 0.11	1.23 ± 0.14	< 0.001^‡^
ApoB (g/L)	0.75 ± 0.15	0.88 ± 0.20	< 0.001^‡^
Fasting blood glucose (mmol/L)	4.72 ± 0.68	5.03 ± 0.58	< 0.001^‡^
Fasting insulin (ng/ml)	7.99 ± 6.64	7.08 ± 3.68	0.135^‡^
HOMA-IRδ	1.75 ± 1.79	1.60 ± 0.85	0.349^‡^
Homocysteine (umol/L)δ	75.9 ± 19.4	124.9 ± 45.0	< 0.001^‡^
C-reactive protein (ng/ml)δ	21.8 ± 8.9	21.0 ± 7.9	0.383^‡^
Albumin (g/L)	49.4 ± 3.5	50.6 ± 2.4	0.001^‡^
Uric acid (mg/dl)δ	334.2 ± 65.7	357.8 ± 77.3	0.005^‡^
γ-glutamyltransferase(IU/L)δ	20.2 ± 13.4	30.4 ± 16.8	< 0.001^‡^
Intima-media thickness (cm)δ	0.59 ± 0.16	0.63 ± 0.10	0.014^‡^

Data are shown as mean ± SD. Paired student's t-test, † For adjusted age; ‡ For adjusted age, BMI, and smoking, drinking, history of diabetes, history of hypertension, and history of hyperlipemia. δAnalysis performed on log-transformed data.

As far as IMT was concerned, there was significantly difference between the two groups (*P *< 0.05). The mean value for the IMT in the right carotid arteries (0.59 ± 0.15 vs. 0.62 ± 0.09 cm) and left carotid arteries, (0.59 ± 0.15 vs. 0.62 ± 0.09 cm) (*P *< 0.05) were calculated. After controlling the age, smoking, drinking, and history of disease, the vegetarians still had thinner IMT as compared to that of the omnivores (data not shown).

### Associations between tertiles of age of starting vegetarian diet and metabolic parameters

The vegetarians were divided into three tertiles according to age of starting the diet (< 17 years, 17 to ≤ 22 years, > 22 years). The TG and HOMA-IR had shown no difference between distinct tertlies of age of starting vegetarian diet (Table 2). Compared to the omnivores, each tertile had significantly lower TC, LDL-C, systolic BP and diastolic BP when controlling for age, even after age, smoking, drinking, duration of vegetarian diet, history of diabetes, history of hypertension, and history of hyperlipemia had been adjusted. Each tertile had significantly lower HDL-C compared to the omnivores, while the HDL-C had showed no difference after adjusting age, smoking, drinking, duration of vegetarian diet, history of diabetes, history of hypertension, and history of hyperlipemia. As far as IMT was concerned, each tertlie of age of starting vegetarian diet had thinner IMT compare to the omnivores, after adjusting for multivariate factors (age, smoking, drinking, duration of vegetarian diet, history of diabetes, history of hypertension, and history of hyperlipemia)

**Table 2:** Metabolic parameters by tertiles of age of starting vegetarian diet.

	Tertiles of age of starting vegetarian diet				P*
		
	Omnivores (n = 129)	Vegetarian < 17 yrs (n = 62)	17 ≦ Vegetarian ≦ 22 yrs (n = 53)	Vegetarian > 22 yrs (n = 56)	
Total cholesterol (mg/dl)	4.89(4.70-5.09)	4.45(4.19-4.72)^†^	4.32(4.09-4.56)^‡^	4.32(4.08-4.56)^‡^	0.002
Triglyceride (mg/dl)	1.38(1.07-1.68)	1.41(1.00-1.83)	1.23(0.86-1.60)	1.32(0.95-1.70)	0.86
HDL-cholesterol (mg/dl)	1.19(1.14-1.25)	1.19(1.11-1.26)	1.16(1.09-1.22)	1.13(1.06-1.20)	0.48
LDL-cholesterol (mg/dl)	3.07(2.89-3.24)	2.63(2.39-2.87)^†^	2.61(2.40-2.82)^‡^	2.57(2.36-2.79)^‡^	0.006
HOMA-IRδ	1.40(0.76-2.04)	2.53(1.66-3.40)	2.17(1.40-2.94)	2.01(1.22-2.79)	0.39
Systolic BP (mmHg)	128(124-132)	114(108-119)^‡^	114(110-120)^‡^	117(112-122)^‡^	0.001
Diastolic BP (mmHg)	79(77-82)	71(67-74)^‡^	71(68-74)^‡^	73(69-76)^‡^	0.003
IMT (cm) δ	0.62(0.60-0.64)	0.57(0.55-0.60)^†^	0.57(0.54-0.59)^‡^	0.58(0.55-0.60)^‡^	0.016
C-reactive protein (ng/ml) δ	18.8(16.9-20.9)	23.3(20.1-27.0)	21.4(18.8-24.3)	17.4(15.3-19.8)	0.050
Homocysteine (umol/L) δ	120.8 (111.8-129.8)	79.2(66.7-91.7) ^‡^	85.9(75.0-96.8) ^‡^	76.6(65.6-87.6) ^‡^	< 0.001

Data are means (95% CL) unless indicated otherwise. P-Value is the difference among three groups using ANOVA test. * Adjusted for age, smoking, drinking, history of diabetes, history of hypertension, and history of hyperlipemia. † *P *< 0.05 vs. Omnivores, ‡ *P*< 0.01 vs. Omnivores. δAnalysis performed on log-transformed data.

### Associations between tertiles of duration of vegetarian diet and metabolic parameters

When the vegetarians were divided according to duration of vegetarian diet (< 6 years, 6 to ≤ 11 years, > 11 years), the vegetarians in each tertile had significantly lower TC, HDL-C, LDL-C, systolic BP and diastolic BP after controlling for age compared to the omnivores (Table 3). The vegetarians also had lower TC, LDL-C and diastolic BP compared to the omnivores when controlling for age, smoking, drinking, history of diabetes, history of hypertension, and history of hyperlipemia. After adjusting for multivariate variables, TG, HDL-C, HOMA-IR and systolic BP had shown no difference between distinct tertlies of the vegetarians and of the omnivores, while only tertile 1(< 6 years) had lower systolic BP and tertile 2 (6 to ≤ 11 years) had lower HDL-C comparing to the omnivores. As for IMT, only the tertile 1 (< 6 years) and tertile 2 (6 to ≤ 11 years) had shown thinner IMT as compared to the omnivores, and the tertile 3 had not shown an advantage of reducing IMT.

**Table 3:** Metabolic parameters by tertiles of duration of vegetarian diet

	Tertiles of duration of vegetarian diet				P*
		
	Omnivores (n = 129)	0 < Vegetarian < 6 yrs (n = 55)	6 ≦ Vegetarian ≦ 11 yrs (n = 61)	Vegetarian > = 12 yrs (n = 55)	
Total cholesterol (mg/dl)	4.95(4.79-5.10)	4.33(4.11-4.54)^‡^	4.39(4.18-4.59)^‡^	4.26(4.02-4.50)^‡^	< 0.001
Triglyceride (mg/dl)	1.39(1.15-1.64)	1.04(0.70-1.37)	1.61(1.29-1.92)	1.26(0.88-1.63)	0.056
HDL-cholesterol (mg/dl)	1.22(1.18-1.26)	1.18(1.12-1.24)	1.11(1.05-1.16)^‡^	1.14(1.07-1.21)	0.026
LDL- cholesterol (mg/dl)	3.09(2.95-3.23)	2.68(2.48-2.87)^‡^	2.55(2.36-2.73) ^‡^	2.55(2.33-2.76)^‡^	< 0.001
HOMA-IRδ	1.71(1.19-2.22)	1.70(0.99-2.41)	2.34(1.68-3.01)	1.97(1.18-2.76)	0.440
Systolic BP (mmHg)	124(121-128)	116(111-121)^‡^	119(115-123)	119(114-124)	0.048
Diastolic BP (mmHg)	78(76-80)	72(69-75)^‡^	73(70-76)^†^	73(70-76)^†^	0.021
IMT (cm)δ	0.62(0.60-0.64)	0.57(0.55-0.59)^†^	0.57(0.55-0.59)^†^	0.60(0.57-0.62)	0.047
C-reactive protein(ng/ml)δ	21.6(20.1-23.1)	22.7(20.2-25.1)	21.9(19.7-24.2)	20.0(17.4-22.6)	0.581
Homocysteine (umol/L)δ	125.4 (119.3-131.5)	80.9(70.9-90.8)	77.9(68.7-87.0)	72.6(62.0-83.1)	< 0.001

Data are means (95% CL) unless indicated otherwise. P-Value is the difference among three groups using ANOVA test. * Adjusted for age, smoking, drinking, history of diabetes, history of hypertension, and history of hyperlipemia. † *P *< 0.05 vs. omnivores, ‡ *P*< 0.01 vs. Omnivores. δAnalysis performed on log-transformed data.

**Figure 1:**
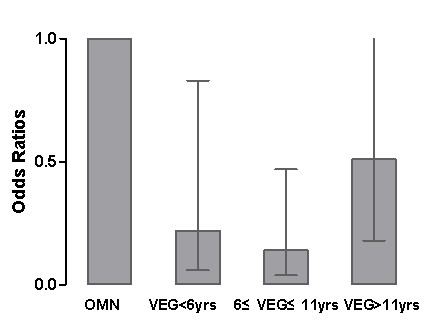
**Risk of cardiovascular disease by tertiles of duration of vegetarian diet**. Adjusted for age, BMI, HOMA-IR, smoking, drinking, history of diabetes, history of hypertension, and history of hyperlipemia. All ORs are statistically significant. OMN, ominivores; VEG, vegetarians.

Correlation analysis showed that duration of vegetarian diet correlated with many metabolic parameters, such as FBG, TC, TG, HDL-C, LDL-C BMI, systolic BP, diastolic BP, HOMA-IR, HCY, CRP, UA and IMT (data not shown). By logistic regression analysis after adjusted for age, FBG, BMI, systolic BP, diastolic BP, HOMA-IR, HCY, CRP, UA, smoking, drinking and history of disease, the odds ratio for IMT in the tertile 2 and tertile 3 were significantly decreased compared to the omnivores (Table 4 and Figure 1). However, these relations after further adjustments for TC, TG, HDL-C and LDL-C disappeared (*P *= 0.140).

**Table 4:** Risk of carotid atherosclerosis according to tertile of duration of vegetarian diet.

	OR (95% CL)				*P*
		
	Omnivores (n = 129)	0 < Vegetarian < 6 yrs (n = 55)	6 ≦ Vegetarian ≦ 11 yrs (n = 61)	Vegetarian > = 12 yrs (n = 55)	
Model 1	1	0.22(0.06-0.83)	0.14(0.04-0.47)	0.51(0.18-1.44)	*0.005*
Model 2	1	0.27(0.07-1.02)	0.18(0.05-0.60)	0.60(0.21-1.73)	*0.025*
Model 3	1	0.34(0.09-1.38)	0.25(0.07-0.92)	0.74(0.24-2.27)	*0.140*

Data are OR (95% CI) unless otherwise indicated. Model 1, Adjustments for age, BMI, HOMA-IR, smoking, drinking, history of diabetes, history of hypertension and history of hyperlipemia; model 2, model 1 + Adjustment for Systolic BP and Diastolic BP, model 3, model 2+ Adjustment for Total cholesterol, Triglyceride, HDL-cholesterol and LDL-cholesterol.

## Discussion

Vegetarian diet had been shown in studies to have beneficial effects on CVD prevention by improving metabolic profile and cardiovascular risk such as blood pressure, lipid profile, insulin sensitivity and vascular dilatory function [3,12,13]. In this study, the vegetarians had thinner IMT compared to the omnivores. Our results demonstrated that the reduction of IMT is dependent on duration of vegetarian diet; however, the effects on reduction of IMT are not significant in those whose duration is beyond 11 years.

Diseases like hypertension and dyslipidemia are important causes of CVD and considered as conventional cardiovascular risk factors. Our data showed that vegetarians had lower systolic BP, diastolic BP and lower serum TC, TG, LDL-C and UA compared to the omnivores. Differences in dietary habits have reflected in these biochemical parameters, which was favorable in vegetarians [14-16]. In the present study, the vegetarians had lower BMI and weight, but larger waist to hip ratio (WHR) than omnivores. Our data supported vegetarian diets were associated with weight loss compared with omnivores [17,18]; in contrast, Chen CW et al. showed that there was no significant difference in BMI and weight between the two groups. High uric acid concentrations are associated with an increased risk of CVD [19]. The high content of nucleic acids in meat results in the formation of more purine-derived uric acid in meat eaters; thus, the lower uric acid concentration in our vegetarian group is expected, this help minimize the risk of CVD.

Dietary patterns modifications had been reported to be associated with reduced circulating levels of acute phase proteins and cytokines [20-22], but they had not been analyzed in parallel for their relative contribution and relevance to reductions in proinflammatory markers. Plasma level of C-reactive protein (CRP) is considered as the predictive value of inflammatory markers on cardiovascular risk [23]; vegetarians probably would not have this value compared to omnivores in our study. Some diabetes prevention studies had shown that lifestyle intervention achieved median reductions in CRP levels [20,24], and Szeto et al. also found that vegetarian diet was associated with lower concentrations of CRP [25]. Actually, weight loss is an important determinant in lowering CRP level [26]. While the weights of the vegetarians was only reduced slightly in the present study, however, the WHRs, a central obesity indicator, were larger in the vegetarians than in the omnivores, and this unfavorable indicator in the vegetarians might counteract the benefit of weight loss on anti-inflammation.

Interestingly, we found markedly lower γ-GT in the vegetarians than in the omnivores. γ-GT is highly related to oxidative stress which plays important roles in both metabolic diseases and CVD. Elevated γ-GT has been considered to be a significant independent predictor of impaired glucose tolerance and diabetes mellitus, and associated with all the features of metabolic syndrome [27]. HCY is an independent risk factor for arterial and venous disease, and a graded association appears to exist between HCY and mortality [28,29]. Mezzano et al. found that mean fasting HCY was higher in vegetarians than in controls [30]; conversely, our study showed that the Chinese vegetarians had lower HCY compared to the omnivores. This finding that supports better metabolic parameters in Chinese vegetarians is likely to predict lower risk of CVD.

Carotid IMT is strongly associated with CVD and its risk factors [31]; therefore it could provide a surrogate end point to assess early atherosclerosis [7,8]. In this study, there were significant differences in carotid IMT between the two groups, IMT in the vegetarians was remarkably thinner than in the omnivores, this is in line with a cross-sectional study which reported long-term low-calorie low-protein vegetarian diet led to significant thinner IMT of carotid arteries [2]. Further analysis showed that reduction of IMT was not dependent on starting age of vegetarian diet but the duration. When the vegetarians were divided into three tertiles according to age of starting vegetarian diet, each tertile showed reducing TC, LDL-C, systolic BP, diastolic BP and carotid IMT. However, the TG, HDL and HOMA-IR showed no difference after adjusting for duration of vegetarian diet, thereby, vegetarians, independent of their age of starting vegetarian diet, have thinner IMT and lower risk of CVD

However, when the vegetarians were divided according to the duration of vegetarian diet, only those in tertile 1 and 2 (< 6 years and 6 to ≤ 11 years) had significantly thinner IMT compared to the omnivores, while tertile 3(> 11 years) did not show beneficial effect. Similarly, each tertile of duration of vegetarian diet has reduced TC, LDL-C and diastolic BP. Logistic regression analysis showed that vegetarian diet played a key role in reducing IMT by improving lipid profile. Few previous studies have discussed the association between duration of vegetarian diet and metabolic cardiovascular risk factor. For vegetarians especially vegans, their diets are deficient in several nutrients including protein, iron, zinc, calcium, vitamin B12 and A, n-3 fatty acids and iodine, this may partly counteract the benefit from this diet habit. Vegetarian diets are also relatively low in α-linolenic acid and provide little eicosapentaenoic acid (EPA) as well as docosahexaenoic acid (DHA) which might exert potent cardioprotective effects [32]. Thus the nutrition status from long-term vegetarian diet may counteract the known cardiovascular health benefits from it. However, the exact explanations remain unclear.

In conclusion, long-term consumption of a low-calorie low-protein vegetarian diet is associated with a decrease in multiple cardiovascular risk factors such as BMI, blood pressure and lipid profile. Moreover, comparing to omnivorous diet, vegetarian diet improves lipid profile that would lead to beneficial effects on carotid IMT, and this appeared to be correlated with the duration of vegetarian diet. However, long-term vegetarian diet, although beneficial on the reduction of IMT, it is deficient in some other nutrients that need to be taken into consideration.

## Competing interests

The authors declare that they have no competing interests.

## Authors' contributions

SYY conceived of the study, involved in the coordination and revised the manuscript. HJZ participated in the design of the study, performed the statistical analysis and drafted the manuscript. SYS performed the statistical analysis and participated in clinical assessment and laboratory measurements. LYW carried out dietary assessment of subjects. BY participated in clinical assessment and measurement of carotid artery IMT. CQL performed clinical assessment and laboratory measurements. WZ participated in clinical assessment and laboratory measurements. XJL participated in its design and coordination. All authors read and approved the final manuscript.

## Supplementary Material

Additional file 1 **Daily dietary intakes of vegetarians and omnivores**. Comparison of dietary components of vegetarians and omnivores. Click here for file
