# Supplementary material for: Relationship of carotid intima-media thickness and duration of vegetarian diet in Chinese male vegetarians
Source: Nutr Metab (Lond). 2011 Sep 19;8:63. doi: 10.1186/1743-7075-8-63 (PMC3184257; doi:10.1186/1743-7075-8-63)
Supplement: Additional file 1 — Daily dietary intakes of vegetarians and omnivores. Comparison of dietary components of vegetarians and omnivores. [file 1743-7075-8-63-S1.DOCX]

Supplemental Table1. Daily dietary intakes of vegetarians and omnivores

| Dietary components | Vegetarians  （n=171） | Omnivores  （n=129） | *P* value |
| --- | --- | --- | --- |
| Energy (kal/d) | 1773.0±502.0 | 2359.5±641.8 | <0.001 |
| Carbohydrate (g/d) | 274.4±102.2 | 340.9±124.5 | <0.001 |
| Protein (g/d) | 53.0±19.3 | 89.9±33.2 | <0.001 |
| Fat (g/d) | 52.7±6.2 | 74.2±16.7 | <0.001 |
| SFA (g/d) | 12.0±4.6 | 21.8±8.3 | <0.001 |
| MUFA (g/d) | 16.3±3.2 | 23.9±7.6 | <0.001 |
| PUFA (g/d) | 24.9±1.4 | 24.7±1.2 | 0.359 |
| Total fiber (g/d) | 17.3±7.2 | 16.6±6.2 | 0.425 |
| Potassium (g/d) | 2.0±0.9 | 2.5±0.9 | <0.001 |
| Sodium (g/d) | 4.1±0.2 | 4.6±0.4 | <0.001 |

All values are means ± standard deviation (SD). SFA, saturated fatty acids; MUFA, monounsaturated fatty acids; PUFA, polyunsaturated fatty acids.
